# Recent Advances in Non-Fullerene Acceptors of the IDIC/ITIC Families for Bulk-Heterojunction Organic Solar Cells

**DOI:** 10.3390/ijms21218085

**Published:** 2020-10-29

**Authors:** Giacomo Forti, Andrea Nitti, Peshawa Osw, Gabriele Bianchi, Riccardo Po, Dario Pasini

**Affiliations:** 1 Department of Chemistry, University of Pavia, Via Taramelli 12, 27100 Pavia, Italy; giacomo.forti01@universitadipavia.it (G.F.); andrea.nitti01@universitadipavia.it (A.N.); peshawa.osw@gmail.com (P.O.); 2 Department of Chemistry, College of Science, Salahaddin University, 44001 Erbil, Iraq; 3 Research Center for Renewable Energies and Environment, Istituto Donegani, Eni Spa, Via Fauser 4, 28100 Novara, Italy; Gabriele.Bianchi1@eni.com (G.B.); riccardo.po@eni.com (R.P.); 4 INSTM Research Unit, University of Pavia, Via Taramelli 12, 27100 Pavia, Italy

**Keywords:** photovoltaics, Bulk-Heterojunction solar cells, acceptor compounds, fused-ring electron acceptors, ITIC

## Abstract

The introduction of the IDIC/ITIC families of non-fullerene acceptors has boosted the photovoltaic performances of bulk-heterojunction organic solar cells. The fine tuning of the photophysical, morphological and processability properties with the aim of reaching higher and higher photocurrent efficiencies has prompted uninterrupted worldwide research on these peculiar families of organic compounds. The main strategies for the modification of IDIC/ITIC compounds, described in several contributions published in the past few years, can be summarized and classified into core modification strategies and end-capping group modification strategies. In this review, we analyze the more recent advances in this field (last two years), and we focus our attention on the molecular design proposed to increase photovoltaic performance with the aim of rationalizing the general properties of these families of non-fullerene acceptors.

## 1. Introduction

Photovoltaic cells are devices that are able to generate electrical power from solar energy. Although various technologies exist, including conventional silicon-based solar cells as well as emerging quantum dot and perovskite solar cells, organic solar cells (OSCs) with bulk heterojunction (BHJ) architecture offer unique advantages for specific applications. For example, OSC panels can be fabricated through low-cost ink-jet processes using environmentally friendly materials and can be scaled up for use in large-area devices. Because they are lightweight and flexible, they could be implemented in non-rigid architectures [1,2,3,4,5,6,7,8,9,10,11,12].

Conventional BHJ-OSCs are typically based on a blend of bicontinuous and interpenetrating electron donor (D) and electron acceptor (A) materials. The absorption of photons creates excitons that dissociate into charge carriers (holes and electrons) at the D/A interface, and the opposite polarity carrier materials transport holes and electrons through the donor and acceptor channels to the anode and cathode, respectively, where they are collected to be used as electrical power source. Efficient exciton dissociation in charge carriers and efficient transport to the electrodes need to be achieved to obtain appreciable power conversion efficiencies (PCEs). Materials need to be chosen to obtain an exergonic driving force for charge separation (ΔG_CS_ < 0) and an efficient migration toward the electrodes.

Typical electron donor polymers exhibit a conjugated backbone on which pendant alkyl side chains, necessary for solubilization in organic solvents and processability, are present. OSCs based on fullerene electron acceptors (FAs) played a central role in the development of increasingly better performing devices, with a maximum PCE currently achieved at 11% [13]. FAs possess several advantages: photostability and efficient light harvesting, easy processability and high electron affinities [14]. 6,6-phenyl C_61_ butyric acid methyl ester (PC_61_BM) and its C_70_-based homologue (PC_71_BM) are the most successful and widely used FAs for solution-processed BHJ OSCs. Their specific advantages can be summarized as follows: (i) strong tendency to accept electrons from common donor semiconductors; (ii) high electron mobility even in the composite form; (iii) ability to form favorable nanoscale morphological networks with donor materials; (iv) isotropy of charge transport; and (v) reversible electrochemical reduction [15]. Record efficiencies in OSCs have been the result of the development of new electron donor materials with improved properties, such as better spectral sensitivity, enhanced hole transport, and more favorable HOMO/LUMO (highest occupied molecular orbital/lowest unoccupied molecular orbital) energy levels, better matching with PC_61_BM or PC_71_BM HOMO/LUMO levels.

More recently, the focus in the development of OSCs has shifted to non-fullerene electron acceptors (NFAs) in combination with a polymeric electron donor in BHJ OSCs. In fact, the PCEs of such cells have increased dramatically since 2015, now reaching a high value of over 16% [16]. An ideal NFA should exhibit (a) strong absorption coefficients in regions of the visible and NIR spectrum that are complementary to those in which the available electron donor polymers absorb; (b) suitably matched energy levels for achieving exergonic charge separation with donors; (c) the ability to form appropriate morphologies for charge separation and suitable percolation pathways for charge transport; and (d) good molecular and morphological thermal and photostability. NFAs have a long history; indeed, the first reported bilayer OSCs used a perylene-based acceptor [17], and some early BHJ OSCs used electron-rich and electron-deficient poly(phenylenevinylene)s [18,19]. A wide variety of material classes have been examined as NFAs [20,21,22,23,24]. Many of the advances in NFA-containing OSCs are attributable to two classes of materials: rylene di-imides and fused-ring electron acceptors (FREAs) [25,26,27,28,29]. By far the best NFA-containing OSCs performances were achieved using FREAs, well-defined organic compounds characterized by a push–pull architecture in which π-extended donating cores with four or more aromatic fused rings are flanked by two electron-withdrawing units (A–D–A molecular structures). Aromatic fused rings have been widely applied in the construction of high-mobility organic semiconductors because the extended conjugation in fused rings is beneficial to forming effective interchain π−π overlaps and enhancing intermolecular charge transport. Typical fused rings have high planarity and suffer from strong aggregation, leading to the formation of large crystalline domains. The introduction of rigid out-of-plane side chains onto the fused rings avoids the formation of large crystalline domains, and it optimizes the exciton diffusion/separation efficiencies. Meanwhile, the introduction of electron-withdrawing moieties (such as imide, amide, and cyano groups) end-capping the π-conjugated core can cause efficient mixing in forming the molecular orbitals, leading to stabilization of the molecular LUMO and reduction of the molecular HOMO–LUMO gap to red-shift absorbance.

**Figure 1:**
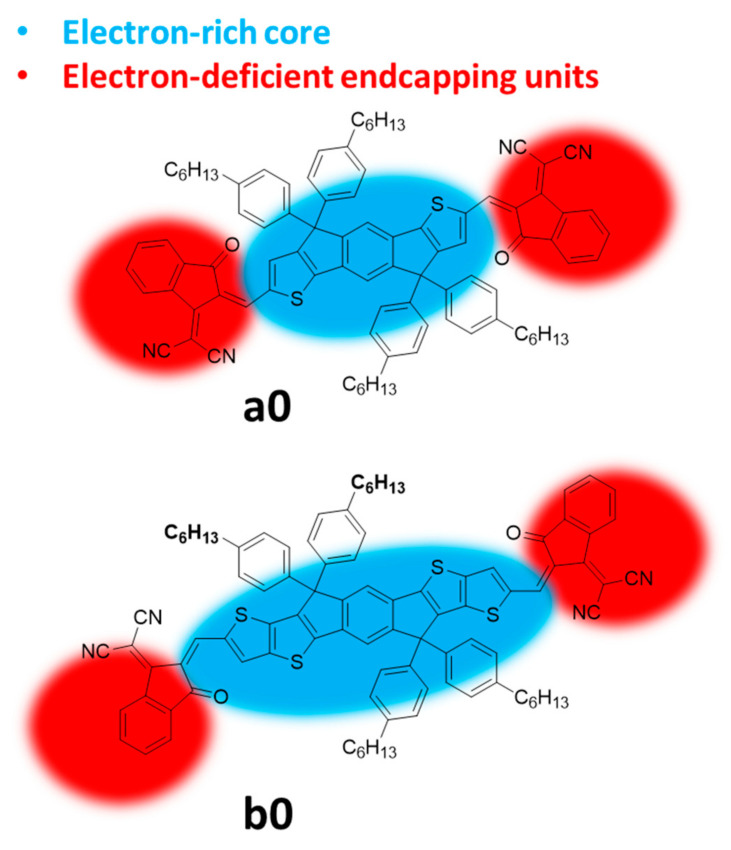
The prototypical member a0 and b0 of the IDIC and ITIC non-fullerene electron acceptors (NFA) families; the areas of possible modifications are highlighted.

Amongst FREAs, the IDIC/ITIC families of NFAs stood out in the last five years in terms of performances. The prototypical IDIC and ITIC molecules, namely, 2,2’-((2Z,2′Z)-((4,4,9,9-tetrahexyl-4,9-dihydro-s-indaceno[1,2-b:5,6-b’]dithiophene-2,7-diyl)bis(methanylylidene))bis(3-oxo-2,3-dihydro-1H-indene-2,1-diylidene))dimalononitrile (IDIC), and 3,9-bis(2-methylene-(3-(1,1-dicyanomethylene)-indanone)-5,5,11,11-tetrakis(4-hexylphenyl)-dithieno[2,3-d:2′,3′-d’]-s-indaceno[1,2-b:5,6-b’]dithiophene) (ITIC), a0 and b0, respectively, are shown in Figure 1. The molecular conjugated cores are decorated by spiro quaternary carbon stereocenters, bearing alkylaryl or alkyl substituents, which have been demonstrated to be essential for intermolecular separation and morphological optimization of the active layer [30,31]. The main differences between the IDIC and ITIC families are the presence of thiophene flanking units with a total of five fused planar aromatic units in the core for the former, and the presence of thienothiophene flanking units with a total of seven fused planar aromatic rings in the latter. We refer to flanking units to indicate a structured extension of the central core and to distinguish them from the endcap groups. Their synthesis is very similar and is comprised of cyclization to form and install the quaternary stereocenters on the preformed core and the final introduction of the electron-deficient end-capping moieties, usually indanedione derivatives, through a condensation reaction.

With respect to other reviews on IDC/ITIC-type materials in the field of organic photovoltaics, we emphasize here the chemist’s point of view, and we report a section on industrial considerations on their synthesis. The field is in continuous evolution, and other families of FREAs [32,33,34,35,36,37,38] have recently demonstrated extremely high potential.

The most effective modifications of the prototypical structures a0 and b0 have been approached at the level of the π-electron rich core and of the π-electron-deficient end-capping moieties (Figure 1). In this review, we illustrate recent advances (in the last 2 years) in the IDIC/ITIC families in order to understand the detailed optoelectronic, morphological, and photovoltaic properties of these acceptor families. Modifications of the lateral solubilizing chains have shown to bring minimal changes to the photovoltaic performances, and such studies are not included. We focus our attention on the rational design of the modifications carried out on the IDIC/ITIC NFAs, and on the outcome in terms of the photovoltaic performance.

## 2. Discussion

### 2.1. Modification of Electron-Deficient End-Capping Groups

**Figure 2:**
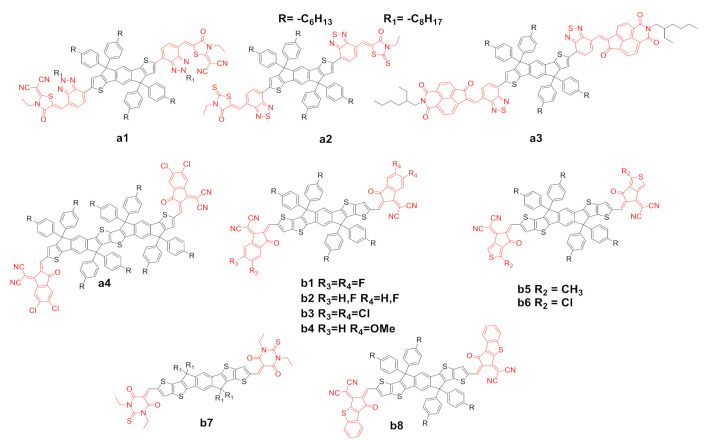
Structure of the of acceptors a1–a4 and b1–b8.

End-capping groups are extremely important for the optical and electrochemical properties of the NFAs for the following reasons: (a) they retrieve electron density from the electron-rich core, generating a push–pull effect responsible for the light absorption in the region of 600–800 nm as well as a spatial confinement of the LUMO orbitals over them [39]; (b) they contribute to the interaction between the acceptors and the donor [40], and to the overall morphological properties of the active layer [41]. Compounds obtained from the formal modification of the basic structure of compound a0 are identified with the letter a, whereas compounds obtained from the formal modifications of the basic structure of compound b0 are identified with the letter b. The most important modifications of the end-capping groups in the last two years are shown in Figure 2, and their properties are summarized in Table 1.

The three main parameters to compare different cells are *J*_SC_, the short circuit current; *V*_OC_, the open-circuit voltage and FF, the fill factor. All three of them need to be maximized to improve the PCE value. Numerous studies have demonstrated that *V*_OC_ depends on the energy gap between the HOMO level of the donor and the LUMO level of the acceptor of the BHJ [42]. *J*_SC_ is primarily dependent on factors related to the efficiencies of each stage in the photovoltaic process, including the efficiency of light absorption, exciton diffusion, exciton dissociation, recombination, charge transport, and charge collection. The FF gives an indication of how easily and what fraction of the generated charges can be removed from a cell and, in the ideal case, will have a value of unity.

Tang et al. [43,44] recently synthesized two novel NFAs, a1 and a2, formally derived from a0. They have similar π-electron-deficient spacers: a benzotriazole for a1 and a benzothiadiazole for a2, as well as different end groups: a1 presents a dicyano-functionalized rhodanine, while a2 has a rhodanine. Absorption spectra of a2 are red-shifted with respect to a1, with an absorption maximum essentially identical to b0. Compound a2 has substantially higher HOMO and lower LUMO levels with respect to a1.

The photovoltaic properties of the best devices for NFAs shown in Figure 2 are reported in Table 2, alongside the indication of the reference and the acronym of the donor polymer used in combination with the NFA for the production of the bilayer cell. A2 showed higher *J*_sc_ compared with a1, presumably as a consequence of a better positioned LUMO, causing a positive difference in terms of PCE (entries 2 and 3 in Table 2). It is noteworthy that a1 has the highest *V*_OC_ reported in Table 2, which, according to the authors, is a result of the optimal energy level matching the used donor polymer.

Kolhe et al. [45] reported earlier this year the synthesis of a novel NFA, a3, which contains the unusual naphthaleneimide mono-ketone as the electron rich end-capping group. A3 showed an absorption maximum at a lower wavelength with respect to a2, demonstrating that the modified naphthalene imide does not possess an optimal electron withdrawing character. Moreover, the absorption coefficient and the electron mobility data are considerably lower than most other NFAs in Table 1. Nevertheless, BHJ cells with a3, using a different donor polymer, gave higher PCE than to a1 and a2. Qu et al. [46] recently reported the synthesis of a novel NFA, a4, formally derived from the substitution of the benzene core of a0 with a thienothiophene unit as central core and chlorinated end groups. A4 showed a remarkable red shift vs. a1–3, while the HOMO/LUMO levels showed a higher energy level for the HOMO, resulting in a lower optical bandgap compared to the previously discussed cases. In terms of photovoltaic properties, a4 did not show an optimal *V*_OC_, but excellent *J*_SC_ resulting in comparable PCEs.

Modifications of the end-capping units of the prototypical ITIC b0 have been reported for a few years by means of di- or tetra-fluorine substitution of the hydrogen of the benzene unit in the modified indanedione terminal units (compounds b1 and b2) [29,47]. Zhang et al. [48] recently reported the synthesis of a novel NFA, b3, which is essentially the chlorinated version of b1. B3 showed a remarkable red shift, a higher dipole moment and a higher intramolecular charge transfer (ICT) with respect to b1. HOMO/LUMO levels are considerably lower in absolute energy compared to those of b1 and b2. The presence of chlorine atoms as suggested by the authors also caused more pronounced molecular stacking, which helps to expand the absorption spectrum and affords very high absorption coefficients. The rather low V_OC_ is largely compensated by high values of *J*_sc_ (see also the high absorbance coefficient) and FF, affording record efficiencies for devices made with b3.

Last year, Li et al. [49] reported the synthesis of a novel asymmetric NFA, b4, which contains two methoxy units in one of the end-capping groups with respect to b0. The authors explain their choice as on this basis of the following: (a) generation of a permanent dipole moment over the whole molecule, with NFA of the kind A1–D–A2; (b) fine tuning of energy levels; and (c) facile and cost-effective synthesis. B4 showed a blue-shifted absorption spectrum (in thin films) with respect to b0 ascribed to the electron-donating capacity of the methoxy groups, resulting in deeper lying HOMO and LUMO levels, but with similar energy gap. In terms of photovoltaic properties, b4, with respect to b0, showed higher V_OC_ as a consequence of the better match with the donor polymer and higher *J*_sc_ and FF (Table 2 entry 10). The authors ascribe this to the presence of the enhanced dipole moment, which enables effective tuning of the molecular binding energy, crystallization properties, and morphology of the blend films, allowing a close molecular packing and an efficient charge transport.

Luo et al. [50,51] reported the synthesis of two novel NFAs b5 and b6, which contain thiophene-fused end-capping groups, differing only in the substituent on the α-position of the terminal thiophene residue, a methyl group for b5 and a chlorine atom for b6. The authors observed markedly different photophysical and photovoltaic properties in these NFAs. B5 and b6 showed an increasing level of red-shifting with respect to b0 and, accordingly, lower HOMO/LUMO energy gaps. In terms of photovoltaic properties b5 and b6, although compared in combination with different donor polymers, gave some of the best performances in the field to date for binary BHJ cells. The authors ascribe the results to balanced hole and electron mobilities, low recombination, more effective carrier extraction and a good morphology of the active layers.

Firdaus et al. [52] reported the synthesis of a novel NFA, b7, which contains 3-diethyl-2-thiobarbituric acid as end groups. B7 is blue-shifted compared to b0; this is ascribed to the different electron withdrawing ability of the end-capping units, which do not allow for optimal push–pull molecular profiles. In any case, photovoltaic efficiencies were over 10%. Last year, Chang et al. [53] reported the synthesis of a novel NFA, b8, which contains a 2-(3-oxo2,3-dihydro-1H-benzo [*b*]cyclopenta[*d*]thiophen-1-ylidene) (α-BC) moiety as the electron-deficient end-capping group. B8 showed similar absorption properties with respect to b0. The α-BC moieties promote a large dipole between the electron-rich benzothiophene and electron-withdrawing dicyanovinylidene moieties, which enhance π–π interactions. In terms of photovoltaic properties, b8 showed better parameters and PCE than those of b0, which, according to the authors, can be attributed to the better packaging and shorter π–π stacking distances.

### 2.2. Structural Modification of the IDIC/ITIC Core

**Figure 3:**
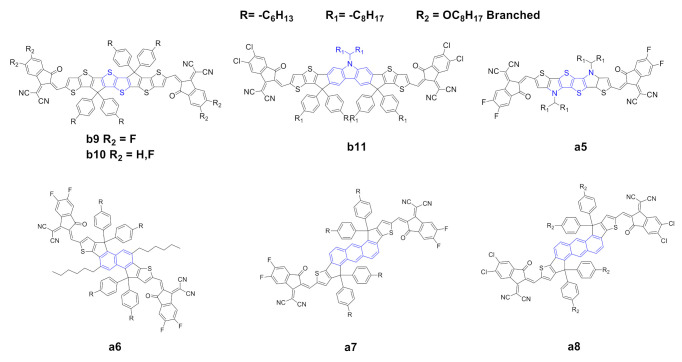
Structure of the acceptors of the IDIC/ITIC families, where the central core of a0 or b0 has been modified.

**Figure 4:**
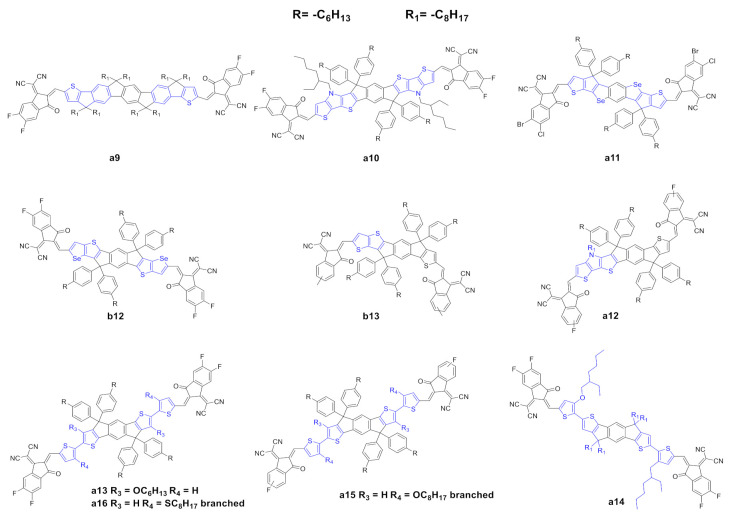
Structure of the of acceptors of the IDIC/ITIC families, where the flanking units of a0 or b0 have been modified.

The modification of the planar conjugated structure is one of the possible strategies to promote fine tuning of the optoelectronic and photovoltaic properties of the IDIC/ITIC families. Many recent reports have investigated the modification of this core by means of variations in the (a) central benzene ring, or (b) lateral thiophene or thienothienophene moieties. The most important modifications to the central core and lateral moieties are shown in Figure 3 and Figure 4. The optical and electrochemical properties are summarized in Table 3, Table 4, Table 5 and Table 6.

Dai et al. [54] reported the synthesis of a novel thienothiophene (TT) core b9. The introduction of the TT moiety increases the electron density and extends the conjugation of the core compared to the classical benzene ring. The UV–vis spectra of b9 showed a remarkable red shift, with higher maximum extinction coefficient, a lower optical bandgap originated from a lower LUMO level and a higher electron mobility with respect to the prototypical ITIC b0. The authors also reported a simultaneously enhancement of *V*_OC_ and *J*_sc_ and, consequently, a higher PCE with respect to b0, other materials and equal parameters (Table 4). Li et al. [55] recently reported the synthesis of a novel NFA, b10, which essentially possesses the same structure as b9 but differs in terms of the end-capping groups: b10 possesses two fluorine atoms instead of four as in the case of b9. B10 has an absorption spectrum with a remarkable red shift due to the halogenated end-capping groups and a higher (almost double) maximum extinction coefficient with respect to ITIC b0; moreover, it has a higher HOMO and a lower LUMO which provoke a lower optical band gap. In terms of photovoltaic properties, b9 and b10 showed higher PCE compared with those of b0; the authors explained this as a consequence of complementary absorption with the donor, higher and more balanced charge mobility and a higher domain purity, which suppresses the bimolecular recombination and promotes charge transport, consequently increasing the PCE.

Chen et al. [56] reported the synthesis of a novel NFA b11 with a carbazole moiety instead of a benzene ring, combined with a chlorine-substituted end-capping group. The carbazole moiety has a strong electron-donating capacity, and, consequently, the resulting core is richer in terms of electron density. Such core substitution, however, gives rise in b11 to substantial red shift, higher HOMO and lower LUMO with respect to b0, and, in addition to the expected lower optical band gap, a higher extinction coefficient at the absorption maxima. In terms of photovoltaic properties, b11 showed higher V_OC_ and FF compared to those of b0, presumably as a consequence, as suggested by the authors, of higher electron/hole mobilities. The carbazole-based acceptor achieved the highest PCE for the binary BHJs reported to date in the literature with this class of NFAs (entry 13 in Table 4). Huang et al. [57] reported the synthesis of a novel NFA, a5, with a tetrathiophene central core in a structure with high planarity, resulting in an extended and electron-rich core compared with that of a0. The visible spectrum of a5 showed a remarkable red shift and a reduced HOMO/LUMO gap. In terms of photovoltaic properties (Table 2, entry 3), a5 showed much higher J_sc_ and FF with respect to a0, which the authors claim to be a result of higher electron mobility and a lower level of charge recombination. Zhu et al. [58] reported the synthesis of a novel NFA with a naphthalene core (a6); the optoelectronic properties are essentially the same as those of a0, apart from higher HOMO and LUMO levels, probably better matching the donor polymer, resulting in better performance in terms of photovoltaic properties. An anthracene moiety was also proposed as a core by Yao et al. [59] and Feng et al. [60] (compounds a7 and a8). These scaffolds present an extension of the conjugation, resulting in a red-shifted UV–vis spectra and good extinction coefficients at λ_max_. HOMO/LUMO energy differences, however, resulted to be higher for a7 and a8 than for a0. In terms of photovoltaic properties, however, a7 and a8 showed better performances when compared to those of a0, with higher values for all relevant parameters, namely, V_OC_, J_sc_, FF and PCE.

The modification of the central core and the corresponding fine tuning of the optoelectronic and photovoltaic properties represent the most important strategy to achieve highly performing IDIC/ITIC NFAs. Modification of the lateral moieties has also been addressed (Figure 4). Liu et al. [61] reported the synthesis of a novel NFA (a9) in which the two thiophene units of a0 were substituted with fluorene-type moieties. This substitution should affect the optoelectronic properties, and the UV spectra is slightly red-shifted with respect to a0 in solution, but such a red shift is not confirmed in thin films (Table 5 and Table 6). Interestingly, a9 possesses a lower electron mobility, but higher photovoltaic parameters than those of a0; the authors explained this as a consequence of better complementary absorption with the donor, suppressed nonradioactive recombination loss, uniform and significantly decreased phase-separated morphology and a more efficiently suppressed charge recombination.

Sun et al. [62] reported the synthesis of a novel NFA, a10, in which the lateral thiophene moieties were substituted with dithienopyrrole moieties. The core extension in a10 allowed a remarkable red shift of the UV spectra to the NIR with a higher maximum molar absorption coefficient; moreover, the introduction of a much more electron rich-core led to a substantially higher HOMO, a lower optical band gap and a higher electron mobility compared with those of a0. In terms of photovoltaic properties, a10 showed better photovoltaic parameters than a0. Wan et al. [63] and Lin et al. [64] reported the synthesis of two novel NFAs, a11 and b12. These acceptors, despite the different halides in the end-capping groups, have a regioisomeric scaffold. A11 and b12 present a remarkable and slightly red-shift UV spectra compared with a0 and b0, respectively, induced by the introduction of the selenium heterocycle. In terms of photovoltaic properties, interestingly, a11 showed higher PCE than b12.

Asymmetric scaffolds represent an interesting strategy to promote fine tuning of the optoelectronic properties and a way to achieve high-performance OSCs. Gao et al. [65] reported the synthesis of a novel NFA, b13, with an asymmetrical scaffold; it can be categorized as an ITIC with a thiophene instead of a thienothiophene. B13 showed basically the same UV spectra with a slightly higher molar extinction coefficient; moreover, b13 has a higher HOMO and a comparable LUMO with respect to b0. Interestingly, the authors observed that the asymmetric scaffold of b13 possessed a higher dipole moment, leading to a high polarity molecule, which could be useful in order to increase the solubility of these molecules in a much more polar solvent than the conventional ones used in device fabrication. In terms of photovoltaic properties, b13 showed higher values for all parameters vs. b0, and consequently higher PCE. The authors explained these results as a consequence of higher and more balanced carrier mobilities, better dissociation probability and charge collection ability, minor bimolecular recombination and excellent morphology. Last year, Yang et al. [66] reported the synthesis of a novel asymmetric NFA, a12, which possesses a dithienopyrrole moiety. A12 showed a remarkable red shift compared to a0, and, in terms of photovoltaic properties, it showed higher values for all parameters vs. a0, and consequently higher PCE.

NFA molecules must be soluble in common organic solvents in order to be processable. Alkyl side chains ensure good solubility in these solvents; the introduction of alkyl chains is, however, associated with other problems such as impeding the ordered packing of molecules, which decreases the charge transport ability, lowers carrier mobility, and deteriorates device performance. Liu et al. [67] synthetized a novel NFA, a13, which bears two hexyloxy groups at its central core in order to modulate the photophysical, photovoltaic and morphological properties. The side chain introduction and the two terminal thiophene rings of a13 provoke a remarkable red shift with respect to a0. In terms of photovoltaic properties, a13 shows higher values for all parameters vs. a0, and, consequently, higher PCE. It is worth noting that the photovoltaic performances were achieved with spin-coated active layers—as stated by the authors, no extra treatment, such as using additives or annealing, was needed. Lee et al. [68] synthetized a novel NFA, a14, which possesses an asymmetrical side chain substitution pattern. A14 showed a remarkable red shifted UV–vis spectrum when compared to a0, while, in terms of HOMO/LUMO levels, a14 showed a lower optical band gap. In terms of photovoltaic properties, it showed higher values for all parameters vs. a0, and consequently higher PCE as a primary consequence of favorable BHJ morphology for efficient charge separation and transport. Lee et al. [69] and Chen et al. [70] expended this work by synthetizing two novel NFAs, a15 and a16, which contain the same side chains but different heteroatoms onto the flanking thiophene rings. Both acceptors showed remarkable red-shifted UV–vis spectra when compared to those of a0. The optical bandgaps are essentially the same for both acceptors. In terms of photovoltaic properties, they showed higher values for all parameters vs. a0, and consequently higher PCE. The authors claim an efficient wavelength collection near NIR, favorable BHJ morphology, well-ordered molecular orientation, and tight face-on packing.

### 2.3. Industrial Considerations and Scalability of NFAs

The cost of fabricating a photovoltaic module entails different contributions, but the higher costs are associated with the active layer materials [71]. The comparison between OSC systems is often made by considering the best PCE of devices, characterizing each specific combination of the polymer donor and acceptor. Although this metric is undoubtedly important, other parameters for the determination of the potential of OSC materials for industrialization must also be considered. The cost of the materials, and, more specifically, of the employed donor and acceptor, is in turn primarily dependent on their synthetic accessibility, which, broadly speaking, is related to the number of required synthetic steps (NSS) [72]. A few years ago, some of us reported quantitative metrics in an attempt to rationalize and quantify the synthetic complexities of donor and acceptor components for OSCs [73]. We calculated the synthetic complexity (SC) data and the cost per gram of material for a series of recent NFAs reported in this review, and the data are reported in Table 7. The synthetic complexities are rather high for all evaluated substrates, essentially as a consequence of the relevant NSS (9–15) required to afford the target molecules. Low values for NSS, such as in the case of entry 2, can result in high costs depending on the costs of the starting materials.

The (photo)stability of solar devices is one of the pillars for the deployment of OPV technology [73]. Guo et al. [74] published a study where a wide range of non-fullerene acceptors was ranked according to their photobleaching rate. They observed that IDIC derivatives are less photodegradable than ITIC derivatives, with optical density (OD) losses per hour lower than 0.3%. For comparison, the OD loss per hour of ITIC is about 12%; derivatives with electron-withdrawing groups on the terminal moieties are more stable, while derivatives with electron-rich substituents are much more prone to degradation. The introduction of an electron-withdrawing benzothiadiazole ring between the indacenodithiophene core and the dicyanoindanone terminals (i.e., the IDTBR family of acceptors, for example, a2) further increases the stability, which makes these materials the most interesting for module manufacturing (providing that the energy level matching the donor is satisfactory) [75]. In any case, the findings described above provide a rule of design of acceptors that may be useful for further OPV material development. The optimal thickness of the active layer of polymer solar cells based on NFAs is typically around 100 nm [76,77], usually attributed to the ideal morphology consisting of a bicontinuous network of small molecule domains of around 15–20 nm. Higher thicknesses generally lead to enhanced bimolecular recombination, low charge mobilities and low FF values. However, two issues exist with these low thicknesses. First, thin films are not able to absorb all photons of the solar spectrum, leading to low *J*sc values. The final PCE is therefore a trade-off between the decrease in FF with the active layer thickness, and the increase in *J*sc. In addition, it is also very difficult for roll-to-roll productions based on printing techniques to reach thin films over a large area in an uniform way. In fact, thin films are very susceptible to film defects (with possible shunts), are not tolerant to thickness variation, and are less reproducible. Ideally, thicknesses of at least 250–300 nm would be required for the scale up. The literature reports a relatively low number of studies (about 50 papers) on devices based on NFA with thick active layers where good efficiencies are maintained. These devices are the result of careful and delicate optimization of the active layer morphology. From this point of view, IDTBR (a2) is again one of the more robust acceptors, with PCE as high as 8.1% for 1010 nm thick devices [78]. IDIC-type acceptors provide devices with 8.5% PCE at a 530 nm thickness (for comparison, the PCE at 105 nm is 12.2%) [79], while in the case of the ITIC-type acceptor, a device with 8.9% at 500 nm has been reported (12.7% at 141 nm) [80].

## 3. Conclusions

In this review, we have discussed recent contributions that appeared in the literature regarding ITIC/IDIC structural modifications. Such modifications, which can be only tentatively and schematically classified into two different categories (core and end-capping group modifications), have brought several advances not only in terms of overall cell efficiencies, but also in terms of understanding of the interplay between the molecular geometry/shape and the performance in the active layer. Some correlations can be identified within the IDIC/ITIC families, indicating which structural features seem to be more promising. Chlorine substitution in the end-capping group, for example, brings about very positive effects, as well as the desymmetrization of the NFA molecule and the introduction of a strong molecular dipole moment. We believe that the trial and error approach, which has traditionally characterized organic photovoltaic research, will benefit from structural design considerations, such as those emerging from this review. The overall stability in the active layer of the IDC/ITIC families of NFAs must be assessed thoroughly before implementation in applied technologies can take place.

## Figures and Tables

**Table 1:** Optical and electrochemical properties of acceptors a0–a4 and b0–b8.

Entry	Compound	T_d_ (°C) ^a^	$\lambda_{max}^{Sol}$ (nm) ^b^	$\lambda_{max}^{Film}$ (nm) ^c^	$E_{g}^{opt}$ (eV) ^d^	ε (λ_max_) (M^−1^·L^−1^) ^e^	HOMO (eV) ^f^	LUMO (eV) ^f^	μ^SCLC^ (cm^2^·V^−1^·s^−1^) ^g^	Ref.
1	a0		664	716	1.62	2.4 × 10^5^	−5.69	−3.91	1.1 × 10^−3^	[30]
2	a1	396	613	636	1.76	1.14 × 10^5^	−5.49	−3.57		[43]
3	a2		662	688	1.68		−5.31	−3.70		[44]
4	a3	354	628	660	1.63	8.8 × 10^4^	−5.38	−3.68	1.44 × 10^−4^	[45]
5	a4	362	715	754	1.43	2.1 × 10^5^	−5.35	−3.91	2.36 × 10^−3^	[46]
6	b0	345	664	702	1.59	1.3 × 10^5^	−5.48	−3.83	3.0 × 10^−4^	[1]
7	b1			717	-	1.16 × 10^5^	−5.66	−4.14	5.05 ×10^−4^	[30]
8	b2		677	720	1.57		−5.67	−4.02	3.26 × 10^−4^	[47]
9	b3			746	1.48	1.4 × 10^5^	−5.75	−4.09		[48]
10	b4		671	695	1.63	1.9 × 10^5^	−5.61	−3.92		[49]
11	b5		674	718	1.58	2.1 × 10^5^	−5.57	−3.92	8.4 × 10^−4^	[50]
12	b6		709	746	1.58	2.3 × 10^5^	−5.58	−4.01	7.8 × 10^−4^	[51]
13	b7		621	654	1.75	2.6 × 10^5^	−5.81	−3.97	1.8 × 10^−4^	[52]
14	b8	433	664	691	1.59	1.5 × 10^5^	−5.49	−3.90		[53]

(^a^) Decomposition temperature; (^b^) λ_max_ in solution; (^c^) λ_max_ in thin films; (^d^) optical band gap in thin films; (^e^) extinction coefficient at λ_max_; (^f^) determined in solution by cyclic voltammetry; (^g^) electron mobility.

**Table 2:** Photovoltaic properties of best devices incorporating NFA acceptors shown in Figure 1.

Entry	Device	Voc (V) ^i^	Jsc (mA·cm^−2^) ^j^	FF (%) ^k^	PCE (%) ^l^	Ref.
1	PDBT-T1:a0	0.89	14.61	63.0	8.19	[30]
2	J61: a1	1.15	10.84	66.17	8.25	[43]
3	PTB7-Th: a2	1.04	13.57	62.64	8.82	[44]
4	PBDB-T: a3	1.04	17.34	60.0	10.08	[45]
5	PBDB-T: a4	0.85	18.9	66.6	10.7	[46]
6	PTB7-TH: b0	0.81	14.21	59.1	6.80	[1]
7	PBDB-T-SF: b1	0.88	20.88	71.3	13.1	[29]
8	PBT1-C: b2	0.90	16.50	70.8	10.54	[47]
9	PBDB-T-2F: b3	0.79	22.67	75.2	13.45	[48]
10	PBDB-T: b4	0.93	18.11	71.52	12.07	[49]
11	J71: b5	0.92	18.41	74.2	12.5	[50]
12	PM6: b6	0.91	20.1	74.1	13.6	[51]
13	PBDB-T: b7	0.98	15.80	69.0	10.08	[52]
14	PBDB-T: b8	0.94	19.90	64.51	12.07	[53]

(^i^) open circuit voltage, (^j^) short-circuit current density, (^k^) fill factor, (^l^) power conversion efficiency.

**Table 3:** Optical and electrochemical properties of acceptors a5–a8 and b9–b11.

Entry	Compound	T_d_ (°C) ^a^	$\lambda_{max}^{Sol}$ (nm) ^b^	$\lambda_{max}^{Film}$ (nm) ^c^	$E_{g}^{opt}$ (eV) ^d^	ε (_max_) (M^−1^·L^−1^) ^e^	HOMO (eV)	LUMO (eV)	μ^SCLC^ (cm^2^·V^−1^·s^−1^) ^f^	Ref.
1	a0		664	716	1.62	2.4 × 10^5^	−5.69	−3.91	1.1 × 10^−3^	[30]
2	a5				1.32		−5.52	−4.11	3.94 × 10^−4^	[57]
3	a6	384	696	730	1.55	1.8 × 10^5^	−5.41	−3.78	1 × 10^−3^	[58]
4	a7		718	778	1.68		−5.69	−4.01		[59]
5	a8		691	709	1.60	2.2 × 10^5^	−5.71	−3.89		[60]
6	b0	345	664	702	1.59	1.3 × 10^5^	−5.48	−3.83	3.0 × 10^−4^	[1]
7	b9	331	788	862	1.27	2.3 × 10^5^	−5.43	−4.00	1.5 × 10^−3^	[54]
8	b10	328	782	836	1.32	2.0 × 10^5^	−5.36	−3.92	1.2 × 10^−3^	[55]
9	b11	338	690	753	1.47	2.3 × 10^5^	−5.72	−4.03		[56]

(^a^) Decomposition temperature; (^b^) λ_max_ in solution; (^c^) λ_max_ in thin films; (^d^) optical band gap; (^e^) extinction coefficient at λ_max_; (^f^) electron mobility.

**Table 4:** Photovoltaic properties of the best devices incorporating NFA acceptors shown in Figure 3.

Entry	Device	V_oc_ (V) ^i^	J_sc_ (mA·cm^−2^) ^j^	FF (%) ^k^	PCE (%) ^l^	Ref.
1	PDBT-T1: a0	0.89	14.6	63.0	8.2	[30]
2	PBDB-T: a5	0.78	23.2	73.0	13.2	[57]
3	FTAZ: a6	0.90	19.7	69.3	12.3	[58]
4	PBDB-TF: a7	0.93	19.0	73.9	13.1	[59]
5	PBDB-TF: a8	0.90	19.5	75.5	13.3	[60]
6	PTB7-TH: b0	0.81	14.2	59.1	6.8	[1]
7	PTB7-Th: b9	0.64	25.1	67.6	10.9	[54]
8	PTB7-Th: b10	0.74	24.0	67.1	12.0	[55]
9	PM6: b11	0.92	22.6	74.0	15.4	[56]

(^i^) Open circuit voltage, (^j^) short-circuit current density, (^k^) fill factor, (^l^) power conversion efficiency.

**Table 5:** Optical and electrochemical properties of acceptors a9–a12 and b12–b13.

Entry	Compound	T_d_ (°C) ^a^	$\lambda_{max}^{Sol}$ (nm) ^b^	$\lambda_{max}^{Film}$ (nm) ^c^	$E_{g}^{opt}$ (eV) ^d^	ε (λ_max_) (M^−1^·L^−1^) ^e^	HOMO (eV)	LUMO (eV)	μ^SCLC^ (cm^2^·V^−1^·s^−1^) ^f^	Ref.
1	a0		664	716	1.62	2.4 × 10^5^	−5.69	−3.91	1.1 × 10^−3^	[30]
2	a9		678	704	1.61		−5.71	−3.96	3.53 × 10^−4^	[61]
3	a10	377	769	821	1.39	3.51 × 10^5^	−5.42	−3.95		[62]
4	a11	350	736	793	1.39		−5.64	−3.95		[63]
5	a12	350	739	775	1.44		−5.51	−3.96		[66]
6	a13		723	765	1.44	9.9 × 10^4^	−5.54	−3.94		[67]
7	a14		790	850	1.28	1.32 × 10^5^	−5.44	−4.15		[68]
8	a15	345	794	839	1.31		−5.34	−4.06		[69]
9	a16	334	751	831	1.30		−5.54	−4.05	2.65 × 10^−4^	[70]
10	b0	345	664	702	1.59	1.3 × 10^5^	−5.48	−3.83	3.0 × 10^−4^	[1]
11	b12		698	752	1.44	2.14 × 10^5^	−5.52	−3.90		[64]
12	b13	300	664	696	1.65	2.00 × 10^5^	−5.60	−3.87	9.64 × 10^−4^	[65]

(^a^) Decomposition temperature; (^b^) λ_max_ in solution; (^c^) λ_max_ in thin films; (^d^) optical band gap; (^e^) extinction coefficient at λ_max_; (^f^) electron mobility.

**Table 6:** Photovoltaic properties of the best devices incorporating NFA acceptors shown in Figure 4.

Entry	Device	Voc (V) ^i^	Jsc (mA·cm^−2^) ^j^	FF (%) ^k^	PCE (%) ^l^	Ref.
1	PDBT-T1: a0	0.89	14.61	63.0	8.19	[30]
2	PBDB-T2F: a9	0.980	17.60	76.0	13.1	[61]
3	PBDB-T: a10	0.852	21.9	69.8	13.1	[62]
4	PM7: a11	0.830	22.91	76.5	14.54	[63]
5	PBDB-T: a12	0.860	22.4	72.4	14.0	[66]
7	PBDB-T: a13	0.850	20.87	72.0	12.79	[67]
8	PTB7-Th: a14	0.74	26.30	67.0	13.1	[68]
9	PTB7-Th: a15	0.817	21.90	65.0	12.1	[69]
10	PTB7-Th: a16	0.750	25.3	69.3	13.2	[70]
11	PTB7-TH: b0	0.81	14.21	59.1	6.80	[1]
12	PBDB-T-2F: b12	0.846	20.21	75.2	13.05	[64]
13	PBDB-T: b13	0.910	16.02	76.8	11.2	[65]

(^i^) Open circuit voltage, (^j^) short-circuit current density, (^k^) fill factor, (^l^) power conversion efficiency.

**Table 7:** Synthetic complexities and relevant parameters for a series of NFAs described in this review.

Entry	NFA	NSS	SC	Cost (EUR/g)
1	b1	12	72	58
2	b0	9	68	113
3	a2	15	97	105
4	a15	15	91	115

## References

[1] Lin Y., Wang J., Zhang Z.-G., Bai H., Li Y., Zhu D., Zhan X. (2015). An Electron Acceptor Challenging Fullerenes for Efficient Polymer Solar Cells. Adv. Mater..

[2] Xu Y.-X., Chueh C.-C., Yip H.-L., Ding F.-Z., Li Y.-X., Li C.-Z., Li X., Chen W.-C., Jen A.K.-Y. (2012). Improved Charge Transport and Absorption Coefficient in Indacenodithieno[3,2-b]thiophene-based Ladder-Type Polymer Leading to Highly Efficient Polymer Solar Cells. Adv. Mater..

[3] Intemann J.J., Yao K., Li Y.-X., Yip H.-L., Xu Y.-X., Liang P.-W., Chueh C.-C., Ding F.-Z., Yang X., Li X. (2014). Highly Efficient Inverted Organic Solar Cells Through Material and Interfacial Engineering of Indacenodithieno[3,2-b]thiophene-Based Polymers and Devices. Adv. Funct. Mater..

[4] Li Y., Yao K., Yip H.-L., Ding F.-Z., Xu Y.-X., Li X., Chen Y., Jen A.K.-Y. (2014). Eleven-Membered Fused-Ring Low Band-Gap Polymer with Enhanced Charge Carrier Mobility and Photovoltaic Performance. Adv. Funct. Mater..

[5] Wang G., Melkonyan S.F., Facchetti A., Marks T.J. (2019). All-Polymer Solar Cells: Recent Progress, Challenges, and Prospects. Angew. Chem. Int. Ed..

[6] Chen L.X. (2019). Organic Solar Cells: Recent Progress and Challenges. ACS Energy Lett..

[7] Yin H., Yan C., Hu H., Ho J.K., Zhan X., So G.L.S.K. (2020). Recent progress of all-polymer solar cells–From chemical structure and device physics to photovoltaic performance. Mater. Sci. Eng. R Rep..

[8] Karunakaran S.K., Arumugam G.M., Yang W., Ge S., Khan S.N., Li X., Yang G. (2019). Recent progress in inkjet-printed solar cells. J. Mater. Chem. A.

[9] Qin J., Lan L., Chen S., Huang F., Shi H., Chen W., Xia H., Sun K., Yang C. (2020). Recent Progress in Flexible and Stretchable Organic Solar Cells. Adv. Funct. Mater..

[10] Ryu H.S., Park S.Y., Lee H.T., Kim J.Y., Woo H.Y. (2020). Recent progress in indoor organic photovoltaics. Nanoscale.

[11] Nitti A., Signorile M., Boiocchi M., Bianchi G., Po R., Pasini D. (2016). Conjugated Thiophene-Fused Isatin Dyes through Intramolecular Direct Arylation. J. Org. Chem..

[12] Nitti A., Bianchi G., Po R., Swager T.M., Pasini D. (2017). Domino Direct Arylation and Cross-Aldol for Rapid Construction of Extended Polycyclic π-Scaffolds. J. Am. Chem. Soc..

[13] Chochos C.L., Katsouras A., Gasparini N., Koulogiannis C., Ameri T., Brabec C.J., Avgeropoulos A. (2017). Rational Design of High-Performance Wide-Bandgap (≈2 eV) Polymer Semiconductors as Electron Donors in Organic Photovoltaics Exhibiting High Open Circuit Voltages (≈1 V). Macromol. Rapid Commun..

[14] Gasparini N., Katsouras A., Prodromidis M.I., Avgeropoulos A., Baran D., Salvador M., Fladischer S., Spiecker E., Chochos C.L., Ameri T. (2015). Photophysics of Molecular-Weight-Induced Losses in Indacenodithienothiophene-Based Solar Cells. Adv. Funct. Mater..

[15] Cai Y., Zhang X., Xue X., Wei D., Huo L., Sun Y. (2017). High-Performance Wide-Bandgap Copolymers Based on Indacenodithiophene and Indacenodithieno[3,2-b]thiophene Units. J. Mater. Chem. C.

[16] Sun H., Liu T., Yu J., Lau T.-K., Zhang G., Zhang Y., Su M., Tang Y., Ma R., Liu B. (2019). A monothiophene unit incorporating both fluoro and ester substitution enabling high-performance donor polymers for non-fullerene solar cells with 16.4% efficiency. Energy Environ. Sci..

[17] Vandewal K., Tvingstedt K., Gadisa A., Inganäs O., Manca J.V. (2010). Relating the open-circuit voltage to interface molecular properties of donor: Acceptor bulk heterojunction solar cells. Phys. Rev. B.

[18] Hou J., Inganäs O., Friend R.H., Gao F. (2018). Organic solar cells based on non-fullerene acceptors. Nat. Mater..

[19] Eastham N.D., Logsdon J.L., Manley E.F., Aldrich T.J., Leonardi M.J., Wang G., Powers-Riggs N.E., Young R.M., Chen L.X., Wasielewski M.R. (2018). Hole-Transfer Dependence on Blend Morphology and Energy Level Alignment in Polymer: ITIC Photovoltaic Materials. Adv. Mater..

[20] Srivani D., Agarwal A., Bhosale S.V., Puvad A.L., Xiang W., Evans R.A., Gupta A., Bhosale S.V. (2017). Naphthalene diimide-based non-fullerene acceptors flanked by open-ended and aromatizable acceptor functionalities. Chem. Commun..

[21] Sung M.J., Huang M., Moon S.H., Lee T.H., Park S.Y., Kim J.Y., Kwon S.K., Choi H., Kim Y.H. (2017). Naphthalene diimide-based small molecule acceptors for fullerene-free organic solar cells. Sol. Energy.

[22] Jina R., Wang F., Guana R., Zhenga X., Zhang T. (2017). Design of perylene-diimides-based small-molecules semiconductors for organic solar cells. Mol. Phys..

[23] Jo J.W., Jung J.W., Wang H.-W., Kim P., Russell T.P., Jo W.H. (2015). Fluorination of Polythiophene Derivatives for High Performance Organic Photovoltaics. Chem. Mater..

[24] Jung W.J., Jo W.H. (2015). Low-Bandgap Small Molecules as Non-Fullerene Electron Acceptors Composed of Benzothiadiazole and Diketopyrrolopyrrole for All Organic Solar Cells. Chem. Mater..

[25] Zhao F., Dai S., Wu Y., Zhang Q., Wang J., Jiang L., Ling Q., Wei Z., Ma W., You W. (2017). Single-Junction Binary-Blend Nonfullerene Polymer Solar Cells with 12.1% Efficiency. Adv. Mater..

[26] Cui Y., Yao H., Gao B., Qin Y., Zhang S., Yang B., He C., Xu B., Hou J. (2017). Fine-Tuned Photoactive and Interconnection Layers for Achieving over 13% Efficiency in a Fullerene-Free Tandem Organic Solar Cell. J. Am. Chem. Soc..

[27] Xie D., Liu T., Gao W., Zhong C., Huo L., Luo Z., Wu K., Xiong W., Liu F., Sun Y. (2017). A Novel Thiophene-Fused Ending Group Enabling an Excellent Small Molecule Acceptor for High-Performance Fullerene-Free Polymer Solar Cells with 11.8% Efficiency. Solar RRL.

[28] Wang J., Wang W., Wang X., Wu Y., Zhang Q., Yan C., Ma W., You W., Zhan X. (2017). Enhancing Performance of Nonfullerene Acceptors via Side-Chain Conjugation Strategy. Adv. Mater..

[29] Zhao W., Li S., Yao H., Zhang S., Zhang Y., Yang B., Hou J. (2017). Molecular Optimization Enables over 13% Efficiency in Organic Solar Cells. J. Am. Chem. Soc..

[30] Lin Y., He Q., Zhao F., Huo L., Mai J., Lu X., Su C.-J., Li T., Wang J., Zhu J. (2016). A Facile Planar Fused-Ring Electron Acceptor for As-Cast Polymer Solar Cells with 8.71% Efficiency. J. Am. Chem. Soc..

[31] Yuan J., Zhang Y., Zhou L., Zhang G., Yip H.-L., Lau T.-K., Lu X., Zhu C., Peng H., Johnson P.A. (2019). Single-Junction Organic Solar Cell with over 15% Efficiency Using Fused-Ring Acceptor with Electron-Deficient Core. Joule.

[32] Fei Z., Eisner F.D., Jiao X., Azzouzi M., Röhr J.A., Han Y., Shahid M., Chesman A.S.R., Easton C.D., McNeill C.R. (2018). An Alkylated Indacenodithieno[3,2-b]thiophene-Based Nonfullerene Acceptor with High Crystallinity Exhibiting Single Junction Solar Cell Efficiencies Greater than 13% with Low Voltage Losses. Adv. Mater..

[33] Zhang Z., Guang S., Yu J., Wang H., Cao J., Du F., Wang X., Tang W. (2020). Over 15.5% efficiency organic solar cells with triple side chain engineered ITIC. Sci. Bull..

[34] Yang C., Zhang S., Ren J., Gao M., Bi P., Ye L., Hou J. (2020). Molecular design of a non-fullerene acceptor enables a P3HT-based organic solar cell with 9.46% efficiency. Energy Environ. Sci..

[35] Asakawa M., Brown C.L., Pasini D., Stoddart J.F., Wyatt P.G. (1996). Enantioselective Recognition of Amino Acids by Axially-Chiral π-Electron Deficient Receptors. J. Org. Chem..

[36] Park S.H., Park S., Lee S., Kim J., Ahn H., Kim B.J., Chae B., Son H.J. (2020). Developement of highly efficient large area organic photovoltaic module: Effects of nonfullerene acceptor. Nano Energy.

[37] Meredith P., Li W., Armin A. (2020). Nonfullerene Acceptors: A Renaissance in Organic Photovoltaics?. Adv. Energy Mater..

[38] Fox D., Metrangolo P., Pasini D., Pilati T., Resnati G., Terraneo G. (2008). Site Selective Supramolecular Synthesis of Halogen Bonded Cocrystals Incorporating the Photoactive Azo Group. CrystEngComm.

[39] Lee J., Ko S.-J., Seifrid M., Lee H., Luginbuhl B.R., Karki A., Ford M., Rosenthal K., Cho K., Nguyen T.-O. (2018). Bandgap Narrowing in Non-Fullerene Acceptors: Single Atom Substitution Leads to High Optoelectronic Response Beyond 1000 nm. Adv. Energy Mater..

[40] Yao H., Ye L., Hou J., Jang B., Han G., Cui Y., Su G.M., Wang C., Gao B., Yu R. (2017). Achieving Highly Efficient Nonfullerene Organic Solar Cells with Improved Intermolecular Interaction and Open-Circuit Voltage. Adv. Mater..

[41] Kim S.W., Lee Y.J., Lee Y.W., Koh C.W., Lee Y., Kim M.J., Liao K., Cho J.H., Kim B.J., Woo H.Y. (2018). Impact of Terminal End-Group of Acceptor−Donor−Acceptor-type Small Molecules on Molecular Packing and Photovoltaic Properties. ACS Appl. Mater. Interfaces.

[42] Elumalai N.K., Uddin A. (2016). Open circuit voltage of organic solar cells: An in-depth review. Energy Environ. Sci..

[43] Tang A., Xiao B., Wang Y., Gao F., Tajima K., Bin H., Zhang Z.-G., Li Y., Wei Z., Zhou E. (2018). Simultaneously Achieved High Open-Circuit Voltage and Efficient Charge Generation by Fine-Tuning Charge-Transfer Driving Force in Nonfullerene Polymer Solar Cells. Adv. Funct. Mater..

[44] Tang L.-M., Xiaob J., Baia W.-Y., Lia Q.-Y., Wanga H.-C., Miaoc M.-S., Yipb H.-L., Xua Y.-X. (2019). End-chain effects of non-fullerene acceptors on polymer solar cells. Org. Electron.

[45] Kolhe N.B., West S.M., Tran D.K., Ding X., Kuzuhara D., Yoshimoto N., Koganezawa T., Jenekhe S.A. (2020). Designing High Performance Nonfullerene Electron Acceptors with Rylene Imides for Efficient Organic Photovoltaics. Chem. Mater..

[46] Qu J., Zhao Q., Zhou J., Lai H., Liu T., Li D., Chen W., Xie Z., He F. (2019). Multiple Fused Ring-Based Near-Infrared Nonfullerene Acceptors with an Interpenetrated Charge-Transfer Network. Chem. Mater..

[47] Li X., Li C., Ye L., Weng K., Fu H., Ryu H.S., Wei D., Sun X., Woo H.Y., Sun Y. (2019). Asymmetric A–D–p–A-type nonfullerene small molecule acceptors for efficient organic solar cells. J. Mater. Chem. A.

[48] Zhang H., Yao H., Hou J., Zhu J., Zhang J., Li W., Yu R., Gao B., Zhang S., Hou J. (2018). Over 14% Efficiency in Organic Solar Cells Enabled by Chlorinated Nonfullerene Small-Molecule Acceptors. Adv. Mater..

[49] Li M., Zhou Y., Zhang J., Song J., Bo Z. (2019). Tuning the dipole moments of nonfullerene acceptors with an asymmetric terminal strategy for highly efficient organic solar cells. J. Mater. Chem. A.

[50] Luo Z., Bin H., Liu T., Zhang Z.-G., Yang Y., Zhong C., Qiu B., Li G., Gao W., Xie D. (2018). Fine-Tuning of Molecular Packing and Energy Level through Methyl Substitution Enabling Excellent Small Molecule Acceptors for Nonfullerene Polymer Solar Cells with Efficiency up to 12.54%. Adv. Mater..

[51] Luo Z., Liu T., Wang Y., Zhang G., Sun R., Chen Z., Zhong C., Wu J., Chen Y., Zhang M. (2019). Reduced Energy Loss Enabled by a Chlorinated Thiophene-Fused Ending-Group Small Molecular Acceptor for Efficient Nonfullerene Organic Solar Cells with 13.6% Efficiency. Adv. Energy Mater..

[52] Firdaus Y., He Q., Lin Y., Nugroho F.A.A., Le Corre V.M., Yengel E., Balawi A.H., Seitkhan A., Laquai F., Langhammer C. (2020). Novel wide-bandgap non-fullerene acceptors for efficient tandem organic solar cells. J. Mater. Chem. A.

[53] Chang S.-L., Hung K.-E., Cao F.-Y., Huang K.-H., Hsu C.-S., Liao C.-Y., Lee C.-H., Cheng Y.-J. (2019). Isomerically Pure Benzothiophene-Incorporated Acceptor: Achieving Improved Voc and Jsc of Nonfullerene Organic Solar Cells via End Group Manipulation. ACS Appl. Mater. Interfaces.

[54] Dai S., Li T., Wang W., Xiao Y., Lau T.K., Li Z., Liu K., Lu X., Zhan X. (2018). Enhancing the Performance of Polymer Solar Cells via Core Engineering of NIR-Absorbing Electron Acceptors. Adv. Mater..

[55] Li T., Dai S., Ke Z., Yang L., Wang J., Yan C., Ma W., Zhan X. (2018). Fused Tris(thienothiophene)-Based Electron Acceptor with Strong Near-Infrared Absorption for High-Performance As-Cast Solar Cells. Adv. Mater..

[56] Chen T.-W., Peng K.-L., Li Y.-W., Su Y.-J., Ma K.-J., Hong L., Chang C.-C., Hou J., Hsu C.-S. (2020). A chlorinated nonacyclic carbazole-based acceptor affords over 15% efficiency in organic solar cells. J. Mater. Chem. A.

[57] Huang C., Liao X., Gao K., Zuo L., Lin F., Shi X., Li C.-Z., Liu H., Li X., Liu F. (2018). Highly Efficient Organic Solar Cells Based on S,N-Heteroacene NonFullerene Acceptors. Chem. Mater..

[58] Zhu J., Ke Z., Zhang Q., Wang J., Dai S., Wu Y., Xu Y., Lin Y., Ma W., You W. (2018). Naphthodithiophene-Based Nonfullerene Acceptor for High-Performance Organic Photovoltaics: Effect of Extended Conjugation. Adv. Mater..

[59] Yao C., Liu B., Zhu Y., Hong L., Miao J., Hou J., He F., Meng H. (2019). Highly fluorescent anthracene derivative as a non-fullerene acceptor in OSCs with small non-radiative energy loss of 0.22 eV and high PCEs of over 13%. J. Mater. Chem. A.

[60] Feng H., Yi Y.-Q.-Q., Ke X., Yan J., Zhang Y., Wan X., Li C., Zheng N., Xie Z., Chen Y. (2019). New Anthracene-Fused Nonfullerene Acceptors for High-Efficiency Organic Solar Cells: Energy Level Modulations Enabling Match of Donor and Acceptor. Adv. Energy Mater..

[61] Liu G., Jia J., Zhang K., Jia X., Yin Q., Zhong W., Li L., Huang F., Cao Y. (2019). 15% Efficiency Tandem Organic Solar Cell Based on a Novel Highly Efficient Wide-Bandgap Nonfullerene Acceptor with Low Energy Loss. Adv. Energy Mater..

[62] Sun J., Ma X., Zhang Z., Yu J., Zhou J., Yin X., Yang L., Geng R., Zhu R., Zhang F. (2019). Side chain engineering on Dithieno[3,2-b:2′,3′-d]pyrrol Fused Nonfullerene Acceptors Enabling Over 13% Efficiency for Organic Solar Cells. Mater. Chem. Front..

[63] Wan S.-S., Xu X., Jiang Z., Yuan J., Mahmood A., Yuan G.-Z., Liu K.-K., Ma W., Peng Q., Wang J.-L. (2020). A bromine and chlorine concurrently functionalized end group for benzo[1,2-b:4,5-b′]diselenophene-based non-fluorinated acceptors: A new hybrid strategy to balance the crystallinity and miscibility of blend films for enabling highly efficient polymer solar cells. J. Mater. Chem. A.

[64] Lin F., Zuo L., Gao K., Zhang M., Jo S.B., Liu F., Jen A.K.-Y. (2019). Regio-Specific Selenium Substitution in Non-Fullerene Acceptors for Efficient Organic Solar Cells. Chem. Mater..

[65] Gao W., Zhang M., Liu T., Ming R., An Q., Wu K., Xie D., Luo Z., Zhong C., Liu F. (2018). Asymmetrical Ladder-Type Donor-Induced Polar Small Molecule Acceptor to Promote Fill Factors Approaching 77% for High-Performance Nonfullerene Polymer Solar Cells. Adv. Mater..

[66] Yang L., Song X., Yu J., Wang H., Zhang Z., Geng R., Cao J., Baran D., Tang W. (2019). Tuning of the conformation of asymmetric nonfullerene acceptors for efficient organic solar cells. J. Mater. Chem. A.

[67] Liu Y., Li M., Zhou X., Jia Q.-Q., Feng S., Jiang P., Xu X., Ma W., Li H.-B., Bo Z. (2018). Nonfullerene Acceptors with Enhanced Solubility and Ordered Packing for HighEfficiency Polymer Solar Cells. ACS Energy Lett..

[68] Lee J., Song S., Huang J., Du Z., Lee H., Zhu Z., Ko S.-J., Nguyen T.-Q., Kim J.Y., Cho K. (2020). Bandgap Tailored Nonfullerene Acceptors for Low-Energy-Loss Near-Infrared Organic Photovoltaics. ACS Mater. Lett..

[69] Lee J., Ko S.-J., Seifrid M., Lee H., McDowell C., Luginbuhl B.R., Karki A., Cho K., Nguyen T.-Q., Bazan G.C. (2018). Design of Nonfullerene Acceptors with Near-Infrared Light Absorption Capabilities. Adv. Energy Mater..

[70] Chen J., Li G., Zhu Q., Guo X., Fan Q., Ma W., Zhang M. (2019). Highly efficient near-infrared and semitransparent polymer solar cells based on an ultra-narrow bandgap nonfullerene acceptor. J. Mater. Chem. A.

[71] Min J., Luponosov Y.N., Cui C., Kan B., Chen H., Wan X., Chen Y., Ponomarenko S.A., Li Y., Brabec C.J. (2017). Evaluation of Electron Donor Materials for Solution-Processed Organic Solar Cells via a Novel Figure of Merit. Adv. Energy Mater..

[72] Machui F., Hösel M., Li N., Spyropoulos G.D., Ameri T., Sondergaard R.R., Jorgensen M., Scheel A., Gaiser D., Kreul K. (2014). Cost analysis of roll-to-roll fabricated ITO free single and tandem organic solar modules based on data from manufacture. Energy Environ. Sci..

[73] Po R., Bianchi G., Carbonera C., Pellegrino A. (2015). “All That Glisters Is Not Gold”: An Analysis of the Synthetic Complexity of Efficient Polymer Donors for Polymer Solar Cells. Macromolecules.

[74] Guo J., Wu Y., Sun R., Wang W., Guo J., Wu Q., Tang X., Sun C., Luo Z., Chang K. (2019). Suppressing photo-oxidation of non-fullerene acceptors and their blends in organic solar cells by exploring material design and employing friendly stabilizers. J. Mater. Chem. A.

[75] Strohm S., Machui F., Langner S., Kubis P., Gasperini N., Salvador M., McCulloch I., Egelhaaf H.-J., Brabec C.J. (2018). P3HT: Non-fullerene acceptor based large area, semi-transparent PV modules with power conversion efficiencies of 5%, processed by industrially scalable methods. Energy Environ. Sci..

[76] Yin A., Zhang D., Cheung S.H., So S.K., Fu Z., Ying L., Huang F., Zhou H., Zhang Y. (2018). On the understanding of energetic disorder, charge recombination and voltage losses in all-polymer solar cells. J. Mater. Chem. C.

[77] Cui Y., Yao H., Zhang J., Zhang T., Wang Y., Hong L., Xian K., Xu B., Zhang S., Peng J. (2019). Over 16% efficiency organic photovoltaic cells enabled by a chlorinated acceptor with increased open-circuit voltages. Nat. Comm..

[78] Whang Z., Liu X., Jiang H., Zhou X., Zhang L., Pan F., Qiao X., Ma D., Ma W., Ding L. (2019). Organic Solar Cells Based on High Hole Mobility Conjugated Polymer and Nonfullerene Acceptor with Comparable Bandgaps and Suitable Energy Level Offsets Showing Significant Suppression of Jsc–Voc Trade-Off. Solar RRL.

[79] Li S., Ye L., Zhao W., Liu X., Zhu J., Ade H., Hou J. (2017). Design of a New Small-Molecule Electron Acceptor Enables Efficient Polymer Solar Cells with High Fill Factor. Adv. Mater..

[80] Zhang L., Zhao H., Li B., Yuan J., Xu X., Wu J., Zhou K., Guo X., Zhang M., Ma W. (2019). A blade-coated highly efficient thick active layer for non-fullerene organic solar cells. J. Mater. Chem. A.

